# Perioperative Myocardial Injury/Infarction After Non-cardiac Surgery in Elderly Patients

**DOI:** 10.3389/fcvm.2022.910879

**Published:** 2022-05-19

**Authors:** Linggen Gao, Lei Chen, Jing He, Bin Wang, Chaoyang Liu, Rong Wang, Li Fan, Rui Cheng

**Affiliations:** ^1^Department of Comprehensive Surgery, General Hospital of Chinese People's Liberation Army and National Clinical Research Center for Geriatric Disease, Beijing, China; ^2^Department of Thoracic Surgery, General Hospital of Chinese People's Liberation Army, Beijing, China

**Keywords:** postoperative myocardial injury, infarction, cardiac troponin, ischemia, treatment strategy

## Abstract

At present, we have entered an aging society. Many diseases suffered by the elderly, such as malignant tumors, cardiovascular diseases, fractures, surgical emergencies and so on, need surgical intervention. With the improvement of Geriatrics, surgical minimally invasive technology and anesthesia level, more and more elderly patients can safely undergo surgery. Elderly surgical patients are often complicated with a variety of chronic diseases, and the risk of postoperative myocardial injury/infarction (PMI) is high. PMI is considered to be the increase of cardiac troponin caused by perioperative ischemia, which mostly occurs during operation or within 30 days after operation, which can increase the risk of short-term and long-term death. Therefore, it is suggested to screen troponin in elderly patients during perioperative period, timely identify patients with postoperative myocardial injury and give appropriate treatment, so as to improve the prognosis. The pathophysiological mechanism of PMI is mainly due to the increase of myocardial oxygen consumption and / the decrease of myocardial oxygen supply. Preoperative and postoperative risk factors of myocardial injury can be induced by mismatch of preoperative and postoperative oxygen supply. The treatment strategy should first control the risk factors and use the drugs recommended in the guidelines for treatment. Application of cardiovascular drugs, such as antiplatelet β- Receptor blockers, statins and angiotensin converting enzyme inhibitors can effectively improve postoperative myocardial ischemia. However, the risk of perioperative bleeding should be fully considered before using antiplatelet and anticoagulant drugs. This review is intended to describe the epidemiology, diagnosis, pathophysiology, risk factors, prognosis and treatment of postoperative myocardial infarction /injury.

## Introduction

With the rapid development of the aging of the world population, there are more and more surgical operations for elderly patients. Elderly patients have low immunity, many perioperative coexisting diseases, degenerative changes in the function of important organs, and the decline of reserve capacity and compensatory capacity. These factors increase the overall risk of major adverse cardiac events when they undergo non cardiac surgery, which brings great pressure to surgeons for the elderly to perform surgery. Elderly surgical patients have significant complication rate and postoperative mortality. Postoperative myocardial injury/infarction (PMI) is a common complication after non cardiac surgery. Recently, it is considered to be the increase of troponin caused by ischemia 30 days or within 30 days after operation, and the short- and long-term mortality are increased (1–6). Most PMI occurs in patients with existing coronary artery disease, mostly due to mismatch of myocardial oxygen consumption/ supply and rupture of coronary plaque (7). Most patients with PMI did not show typical symptoms of myocardial ischemia such as chest pain, dyspnea and angina pectoris; and only about 20–40% have ischemic features (such as electrocardiography abnormalities and ischemic symptoms). Therefore, PMI in elderly patients is often ignored by clinicians. However, the mortality of patients with asymptomatic PMI is almost as high as those with symptoms (6, 8).

## Epidemiology

There are approximately 300 million non-cardiac surgeries worldwide every year (9). Nearly 5% of non-cardiac surgery patients will have adverse cardiovascular complications 30 days or within 30 days after operation, mainly including myocardial infarction / injury, cardiac death, cardiac arrest and so on (8). The cardiovascular complications are still the main factors leading to perioperative morbidity and mortality (10). The incidence of PMIs in the non-cardiac surgery patients ranged from 3.5% (11) to 19.1% (12). The results of an international prospective cohort of 15,065 hospitalized patients aged 45 or over who underwent non cardiac surgery showed that 1,200 patients (8.0%) developed min, of which 58.2% did not meet the universal definition of myocardial infarction, and only 15.8% of patients with PMI had ischemic symptoms. A systematic review and meta-analysis showed that the incidence of PMI was 17.9% (9). Multivariate analysis showed that over 75 years old, hypertension, heart failure, coronary heart disease, diabetes, and end-stage renal failure were the risk factors for PMIs. The researchers also developed a model to predict 30 day mortality in patients with PMI. Age older than 75 years, ST segment elevation or new left bundle branch block or anterior ischemic ECG changes were independent predictors of 30 day mortality in patients with PMI (8).

## Diagnosis

The diagnostic criteria of PMI come from a large number of epidemiological studies through multivariate analysis. It is recommended to routinely screen the level of troponin in elderly patients undergoing non cardiac surgery. The diagnostic criteria are as follows: the increase of troponin within 30 days after operation is judged as myocardial ischemia, with or without chest pain, precordial discomfort, ECG changes and other characteristics of myocardial ischemia (6, 8, 13). The Vascular events In non-cardiac Surgery patIents cOhort evaluatioN (VISION) study is the largest prospective cohort study evaluating PMI at present. The troponin threshold of PMI is determined as: (i) high sensitivity troponin T (hsTnT) is 20–65 ng / L, and the absolute change is at least 5 ng / L; and (ii) a non-high-sensitivity troponin T >30 ng/L (5, 14). A prospective diagnostic multicenter study demonstrated that using high sensitivity cardiac troponin I (hs-cTnI), PMI was less common vs. using hs-cTnT. Using hs-cTnI, PMI remained independent predictor of 30-day and 1-year mortality. Therefore, PMI diagnosed by hs-cTnI also has important prognostic significance (15).

Because the clinical symptoms of PMI are not typical, it is difficult to identify PMI according to the clinical symptoms. Routine perioperative troponin monitoring is an important method to identify patients with PMI. The Canadian Cardiovascular Society guidelines recommend that patients with cardiovascular risk > 5% should be monitored for troponin screening before operation and continue to be screened within the first 3 days after operation (16). The European Society of Cardiology (ESC) / European Society of Anesthesiology (ESA) and American College of Cardiology / American Heart Association guidelines also recommend routine evaluation of cardiac troponin during perioperative period.

## Pathophysiology

The pathophysiology of PMI is multifactorial and remains debate. The Fourth Universal Definition of Myocardial Infarction provides a classification of acute myocardial injury, including 5 subtypes of MI and non-ischemic myocardial injury. PMI is a complex syndrome with many different potential causes, including type 1 myocardial infarction (T1MI) and type 2 myocardial infarction (T2MI). The former emphasizes the causal relationship between plaque destruction and coronary atherosclerotic thrombosis; the latter has nothing to do with acute coronary atherosclerotic thrombosis and is related to the imbalance of oxygen supply and demand (17).

Surgical trauma and invasive operation related to general anesthesia leads to acute systemic inflammatory reaction, resulting in the increase of myocardial oxygen demand, which is one of the main causes of PMI. The inflammatory states increase C-reactive protein levels, the plasma tumor necrosis factor-alpha, interleukin (IL)-1 and IL-6 (18–20). The inflammatory factors mentioned above trigger and exacerbate ischemic heart injury, small vessel obstruction embolism, thrombosis, and watershed infarction (T1MI). However, PMIs after non-cardiac surgery are apparently largely considered type 2 infarction (21, 22). The pathophysiology of T2MI reflects myocardial ischemia caused by increased myocardial oxygen demand and / or decreased myocardial oxygen supply. It can be seen that the pathophysiological process of PMIS after non cardiac surgery is obviously more consistent with T2MI. A large number of studies have shown that most perioperative cardiac events occur in the first 7 days after operation. The OPTIMUS and Coronary CTA VISION Studies showed that At least 70 to 75% may be due to the imbalance of myocardial oxygen supply and demand, while at most 25 to 30% of PMIS is due to thrombosis (1, 23, 24).

## Prediction by Risk Factors of PMI

PMI usually has no symptoms such as chest tightness, chest pain, precordial discomfort. If troponin is not routinely monitored, it is not easy to be diagnosed. It is increasingly recognized that the high-risk patient for PMI can be identified before surgery. The main causes result in significant myocardial oxygen supply/demand mismatch after surgery include the preoperative, intraoperative and postoperative risk factors. Patients with the following characteristics are prone to PMI: (i) male sex; (ii) age ≥ 75 years; (iii) multi-comorbidities (coronary artery disease, chronic heart failure, peripheral arterial disease, hypertension, stroke, renal insufficiency, diabetes et al.); and (iv) urgent or emergent surgery.

## Age

Frailty, cognitive impairment, comorbidities increase markedly with age. Age is an independent predictor of cardiovascular disease, diabetes and cerebrovascular disease (25), which increase overall risk for major adverse cardiac events when the elderly patients undergo non-cardiac surgery. Aging and comorbidities are the main risk factors for reducing the tolerance of elderly patients to surgical stress (26). Although the perioperative mortality of elderly patients with acute myocardial infarction is higher than that of young patients, age is not an independent predictor of perioperative cardiac risk.

## Previous Coronary Artery Disease

Patients with a past history of coronary artery disease deserve special attention because it is a powerful predictor of PMI. Major adverse cardiac events after non-cardiac surgery are often associated with prior coronary artery disease. A study showed that patients with unstable angina had increased incidence of perioperative morbidity and mortality (27). With the extension of the time from myocardial infarction to operation, the postoperative myocardial infarction rate and 30-day mortality decreased significantly (28). Large prospective studies have shown that 11–21% of perioperative myocardial infarction is ST segment elevation myocardial infarction (STEMI), and thrombosis exists in almost all STEMIs (1, 24, 29). The OPTIMUS Study showed that 23–31% of perioperative myocardial infarction is caused by coronary thrombosis (22).

The VISION Study prospectively included 955 patients undergoing non cardiac surgery. All patients underwent coronary computed tomography (CT) angiography before operation. The results showed that 96% of 71 patients (7%) with perioperative myocardial infarction showed obstructive or extensive obstructive coronary artery disease (23).

## Intraoperative Risk Factors

Intraoperative risk factors for PMI conclude as the following: open surgery (vs. endovascular surgery), prolonged intraoperative time with hypotension, intraoperative heart rate of >110 or <55 beats per minute, tachycardia, intraoperative transfusions, and receive perioperative vasopressors than patients without myocardial injury after non-cardiac surgery (1, 8, 30–34). Intraoperative hypotension has been shown to be a very important risk factor for the development of myocardial injury (35). The longer duration of intraoperative hypotension (Mean Arterial Pressure < 55 mmHg) is an independent risk factor for PMI (33, 35).

## Postoperative Risk Factors

Postoperative bleeding, sepsis, hypoxia, sustained tachycardia, hypotension, severe anemia is associated with PMIs (13, 36, 37). After adjusting for baseline factors, in patients with postoperative anemia (hemoglobin concentration < 110 g L-1), the risk of PMI increased 1.46 times for every 10 g L-1 reduction in postoperative hemoglobin concentration (95% confidence interval: 1.37–1.56; *P* < 0.001) (37). Treating anemia with transfusions remains controversial. Transfusions might increase fluid overload, the cardiac burden, immunosuppression, and mortality of surgical patients. There is a high rate of anemia and blood transfusion in older patients (38). A restrictive strategy to maintain hemoglobin was between 70 and 90 g L^−1^ and a liberal strategy was between 90 and 110 g L^−1^. The optimum transfusion threshold is not known. Large studies of restrictive vs. liberal transfusion practice with cardiac adverse events in elderly patients is warranted.

## Perioperative Risk Assessment

Elderly patients at risk of cardiovascular adverse events should undergo a thorough perioperative risk assessment. First of all, the clinicians should inquire about the medical history in detail and carry out physical examination in detail. Then, perioperative risk assessment usually uses a combination of non-invasive cardiac testing, clinical risk prediction models, and cardiac biomarkers. Three validated risk-prediction tools have been recommended to predict perioperative non-cardiac surgery: the revised cardiac risk index (RCRI), American College of Surgeons National Surgical Quality Improvement Program (NSQIP) Myocardial Infarction and Cardiac Arrest (MICA) index (39), and American College of Surgeons NSQIP Surgical Risk Calculator (40). The RCRI is a validated, simple, and accepted tool to assess perioperative risk of major cardiac complications. RCRI has six predictors of risk as the following: creatinine ≥ 2 mg/dL, heart failure, insulin-dependent diabetes mellitus, intrathoracic, intra-abdominal, or suprainguinal vascular surgery, History of cerebrovascular accident or TIA, Ischemic heart disease. Patients with more predictors of risk would have higher risk. Although RCRI has a good distinction between high-risk and low-risk mace patients after non-cardiac surgery, it is poor in predicting cardiac events after vascular surgery or all-cause mortality after non-cardiac surgery.

The developers of the American College of Surgeons NSQIP MICA tool included 211,410 surgical patients from more than 250 hospitals to form the database. The types of surgery include non-cardiac surgery such as aorta / peripheral blood vessels, chest / abdominal cavity, brain surgery, gynecology and orthopedics, as well as cardiac surgery. NSQIP MICA tool consists of five factors, including operation type, age, dependent functional status, American Association of anesthesiologists (ASA) grade and abnormal creatinine (>1.5 mg / dl). Its perioperative mace is defined as myocardial infarction or cardiac arrest within 30 days after operation. NSQIP MICA index is presented online in the form of interactive table, which is easy to apply. Clinicians only need to input the clinical data of corresponding patients online to obtain the perioperative mace probability calculated based on the database, so as to help doctors and patients clarify the operation risk / benefit ratio and simplify the process of informed consent.

American College of Surgeons NSQIP Surgical Risk Calculator can predict death, death within 30 days after operation, myocardial infarction and venous thromboembolism, which is based on data extracted from 1.4 million operations. The calculator is not only a convenient one-stop comprehensive postoperative risk assessment, but also includes patient-centered prognosis assessment. The performance of NSQIP surgical risk calculator in predicting adverse cardiac events is better than RCRI, but the main defects are: (i) underestimate the actual risk of perioperative myocardial infarction, because the definition of central myocardial infarction is only based on ECG changes, ST segment elevation or new left bundle branch block; (ii) In order to use NSQIP tool to calculate risk, in addition to 21 data elements, CPT code is also required (authorization is required); (iii) The all English interface and the dependence on the network environment affect its ease of use for non-native English speaking countries.

A preoperative NT-proBNP/BNP measurement, resting echocardiography, functional capacity test is useful to predict PMI (41). Postoperative troponin monitoring in elder patients may help reduce postoperative cardiovascular events.

## Prognosis Significance of PMI

A prospective cohort study recruited 21 842 participants aged over 45 years who underwent non-cardiac surgery at 23 centers in 13 countries. The results of this study showed that PMI was associated with an increased risk of 30-day mortality (adjusted HR, 4.69; 95% CI, 3.52–6.25) (6). A large international prospective cohort study showed the prognostic effect of PMI, patients with PMIs were at higher risk of congestive heart failure, a non-fatal cardiac arrest, stroke and 30-day mortality rate compared with non-PMI group (8). In a large retrospective clinical study, 51,071 patients with non-cardiac surgery were selected. The results were consistent with the results of VISION study. It was found that PMIs was associated with increased risk and a decreased time to death (42). In addition, a systematic review and meta-analysis of 530,867 patients undergoing non cardiac surgery in 169 studies showed that patients with PMIs had higher overall postoperative and 1-year postoperative mortality than patients without PMIs (9).

## Management

The complexity of perioperative management of the elderly patients with multiple comorbidities has been recognized, and the challenge is great. The perioperative management strategy of PMI should be multi-dimensional. Firstly, conduct a comprehensive perioperative evaluation, identify high-risk patients of PMI, improve risk factors in time, design with the prevention strategies, and minimize the risk of PMI. Secondly, routine screening of troponin during perioperative period, timely diagnosis of PMI, and recommend targeted clinical treatment according to clinical experience and guidelines as soon as possible to improve the prognosis of patients. Individualizing care in elderly patients with multi-morbidities are very important. Perioperative close monitoring of fluid balance, avoidance of myocardial ischemia, administration of cardiac protecting drugs, and timely treatment of elderly patients with sepsis are particularly important.

The primary prevention plans are firstly focused around the risk factors and guideline-directed medical therapy such as smoking cessation, antiplatelet for coronary, antihypertension therapy, antihyperlipidaemic medications, glycaemic control, anticoagulation for atrial fibrillation. Especially cardiovascular medical treatments, such as antiplatelets, β-blockers, statins, and angiotensin-converting enzyme inhibitors have been shown to improve outcomes in patients with PMIs.

The results of POISE Trial showed that patients with PMIs could benefit from aspirin and statin therapy (1). Aspirin administration can reduce 30-day mortality. However, aspirin was associated with increased bleeding. Given these conditions, physicians should give consideration for weighing the benefits of preventing PMI by using antiplatelets and anticoagulations against the surgical risk of bleeding. Oral statins can significantly improve the long-term prognosis of patients with PMI (43). Based on these clinical studies, aspirin and statins are recommended in patients with PMIS to improve the prognosis.

For patients with PMI, treatment of supply/demand mismatch is very important. For every 10 beats / min increase in baseline heart rate, the risk of PMI is greatly increased (1). When contraindications (such as hypotension, bradycardia and acute heart failure) are excluded, early initiating β-blockers to decrease heart rate and control high myocardial demand should be considered after surgery. A large observational study suggested continuing chronic β-blocker therapy decreases perioperative mortality (44). A systematic meta-analysis demonstrated that β-blockers therapy initiated within 24 h of non-cardiac surgery decreased the incidence of non-fatal myocardial infarction. However, it increased the risk of hypotension, bradycardia, stroke and death (41).

At present, there is a lack of sufficient evidence-based evidence to support whether patients with PMI can undergo perioperative coronary angiography and interventional intervention. Multidisciplinary teams should make individualized decisions according to the patient's condition. One large database study that included a propensity-matched cohort of 34,650 patients who had a myocardial infarction after non-cardiac surgery. The results of this study showed that a coronary angiography management strategy was associated with lower in-hospital mortality than a medical management strategy [8.9 vs. 18.1%, respectively; odds ratio (OR) 0.44; 95% CI 0.41–0.47] (24). However, for patients at risk of bleeding during perioperative period, the benefits and risks of coronary intervention should be weighed more carefully, because antiplatelet drugs need to be taken routinely after stent implantation to prevent thrombosis in stent. However, the use of antiplatelet drugs can significantly increase the risk of bleeding (23).

## Conclusions

The concept of PMI can be simply summarized as follows: it refers to myocardial injury/infarction occurring within 30 days or during non-cardiac surgery, with or without ischemic symptoms due to ischemia. It is one of the most common major perioperative vascular complications and is closely related to short-term and long-term adverse clinical outcomes. Therefore, troponin screening after surgery is recommended in the elderly. Individualizing perioperative assessment and surgery-specific cardiovascular evaluation in elderly patients with multi-morbidities are very important. Management strategy includes modifications of the risk factors and cardiovascular medical treatments. The primary prevention plan includes identifying and modifying risk factors, maintaining perioperative fluid, electrolyte and acid-base balance, appropriately controlling heart rate, reducing myocardial oxygen consumption, avoiding myocardial ischemia, taking appropriate heart protective drugs, timely treating sepsis, etc. Cardiovascular medical treatments include antiplatelets, β-blockers, statins, and angiotensin-converting enzyme inhibitors. Physicians should fully weigh the benefits of preventing PMI by using antiplatelets and anticoagulations against the surgical risk of bleeding.

## Author Contributions

LG, LC, and JH conceived the idea for the study and developed the search strategy. LG, BW, CL, and RW summarized the references. LF and RC had full access to the manuscript and were responsible for the decision to submit the manuscript for publication. All authors contributed to the writing of the manuscript.

## Funding

This work was supported by the Military Healthcare Fund 19BJZ35.

## Conflict of Interest

The authors declare that the research was conducted in the absence of any commercial or financial relationships that could be construed as a potential conflict of interest.

## Publisher's Note

All claims expressed in this article are solely those of the authors and do not necessarily represent those of their affiliated organizations, or those of the publisher, the editors and the reviewers. Any product that may be evaluated in this article, or claim that may be made by its manufacturer, is not guaranteed or endorsed by the publisher.
